# Editorial: Genomics of Lymphoproliferative Disease

**DOI:** 10.3389/fonc.2021.660016

**Published:** 2021-03-17

**Authors:** Francesco Maura, Luca Agnelli, Stefania Bortoluzzi

**Affiliations:** ^1^ Myeloma Program, Sylvester Comprehensive Cancer Center, University of Miami, Miami, FL, United States; ^2^ Myeloma Service, Department of Medicine, Memorial Sloan Kettering Cancer Center, New York, NY, United States; ^3^ Department of Pathology, IRCCS National Cancer Institute, Milan, Milan, Italy; ^4^ Department of Molecular Medicine, University of Padua, Padua, Italy

**Keywords:** lymphoma, leukemia, multiple myeloma, lymphoproliferative disease, transcriptome analysis, gene mutation analysis, integrative “omics”

In the last years, next generation sequencing (NGS) technology has progressively revolutionized clinical and research approaches either to solid or hematological cancers. Taking advantage of an unprecedented resolution, scientists are currently able to comprehensively characterize several genomic and transcriptomic aspects of the cancer cell with both diagnostic and prognostic relevance. Recent studies have proposed different genomic classifiers to sharply stratify distinct tumor entities and define novel genetic drivers of cancer progression and resistance to current therapies. Our appreciation of the transcriptome complexity and the role of small, long and circular RNAs in disease mechanisms is continuously increasing, expanding the repertoire of potentially druggable targets. Indeed, several relevant and fascinating challenges are about to be addressed in the coming years: identifying the functional and biological significance of the driving genetic events; translating fundamental science into daily diagnostic clinical practice; finding novel prognostic markers and therapeutic targets to ultimately implement precise, effective and cost-saving therapies for patients with leukemia or lymphoma tumors.

In the Research Topic “Genomics of Lymphoproliferative Disease”, we collect the contributions to the field from outstanding Researchers investigating lymphoid malignancies with “-*omics*” tools.

Diffuse large B-cell lymphoma (DLBCL) represents the most common histotype of lymphoma and to date remains still incurable for a large fraction of patients. A deeper characterization of DLBCL is the basis of a better knowledge of the disease for patients’ care. In their Review, Cascione et al. highlighted the new molecular classifications of diffuse large B cell lymphomas, with emphasis on copy number variation. The Authors analyze two specific aspects, namely the transformation from indolent lymphoma and diffuse large B cell lymphoma in immunodeficient patients, providing the scientific community with a useful and essential overview of the major molecular classifications described so far. Opinto et al. explored the opportunities offered for the functional characterization of the tumor microenvironment in lymphoma pathogenesis, highlighting the translational value of “-omics” investigations in DLBCL and examining how the interplay between mesenchymal and hematopoietic counterparts in secondary lymphoid organs governs tumor evolution, with peculiarities of different disease subtypes and relevant prognostic implications.

A perspective on the role in Anaplastic Large Cell Lymphoma (ALCL) of small RNA (sRNA) cargos transported by circulating extracellular vesicles was provided by Lovisa et al. There is high interest in studying exosomes of cancer patients both to develop non-invasive liquid biopsy tests for risk stratification and to elucidate their possible involvement in disease mechanisms. RNA-seq profiling of the content of circulating exosomes of ALCL pediatric patients and healthy controls disclosed that non-miRNA derived sRNAs constitute a prominent fraction loaded in exosomes and identified those sRNAs significantly more abundant in exosomes of ALCL patients than in controls. Further investigation identified *RNY4* massive loading into exosomes of ALCL patients, particularly those with advanced and aggressive disease. In ALCL, emerging data indicate that circulating YRNA can reflect the biology of the tumor or the patient’s immune system reaction and prompt a further study of *RNY4* involvement in ALCL tumor microenvironment and disease aggressiveness. A novel oncogenic fusion gene *MEIS1*–*FOXO1* was described in pediatric B-cell acute lymphoblastic leukemia (ALL), showing that a reduced *FOXO1* expression is associated with unfavorable chemoresistant ALL subtype at high risk of relapse (Zheng et al.). Fattizzo et al. provided a focus on the physiopathology of T-cell acute lymphoblastic leukemia (T-ALL). Despite recent significant progress, the biology and the molecular drivers responsible for T-ALL aggressive clinical courses are not fully deciphered. Considering the large availability of new therapies (e.g. small molecules, CAR-T and monoclonal antibody), the Authors discussed the importance of molecular aspects with clinical, prognostic, and therapeutic significance.

Multiple myeloma (MM) was the subject of several articles, particularly in relation to the opportunities to improve disease monitoring offered by genomics. The critical importance of minimal residual disease (MRD) evaluation in MM was examined (Oliva et al.). The integration of MRD assessed by next-generation flow cytometry or next-generation sequencing is emerging as a key endpoint for the multiple myeloma community. In this review, authors addressed many questions regarding the future clinical use of MRD. The excellent review of Bolli et al. tackled the feasibility of NGS-based approaches for detailed genomic and transcriptomics characterization of MM in individual patients, to achieve an effective personalized treatment and management. The benefits and pitfalls of NGS-based diagnostics are analyzed in the paper, highlighting crucial aspects that must be considered for diffuse implementation. In MM, routine clinical markers can capture a few main high-risk features but are “blind to” most genomic lesions, that are particularly numerous and highly heterogeneous in this malignancy. Moreover, available evidence of clonal evolution in MM, with changes in sub-clonal structure and enrichment of high-risk features is pushing the clinics to the use of NGS-based methods to collect molecular data more informative also of disease evolution and resistance. Escape of MM to currently available therapies is the focus of Manni et al., who focus on MM cell ability to adapt to different chronic stress factors and discuss actionable molecules and pathways related to cellular fitness and stress resistance that can be targeted to treat the disease. High-throughput studies, such as structural genomics and transcriptomics, indicated potential vulnerable targets of MM biology. Additionally, major evidence obtained by RNAi, CRISPR/CAS9 and high throughput drug screenings highlighted novel putative regulators of MM cell survival and resistance to stress that could be therapeutically targeted.

Other original articles provide interesting updates also on less frequent entities, highlighting the most recent discoveries and discussing largely unknown aspects of their pathogenesis. Fattizzo and Barcellini summarize what we know about the connection between chronic lymphocytic leukemia and autoimmune complication. Ferla et al. explored the biology of post-transplant lymphoproliferative disorders (PTLD), a rare complication whose genomic background remains largely unknown. Finally, Teramo et al. provide a detailed summary of the genetic lesions involved in Large Granular Lymphocyte Leukemia pathogenesis.

## Author Contributions

FM, LA and SB equally contributed to draft and revise the editorial. The missing Topic editors have read and approved the manuscript. All authors contributed to the article and approved the submitted version.

## Funding

This work has been supported by the Sylvester Comprehensive Cancer Center NCI Core Grant (P30 CA 240139) and by the Memorial Sloan Kettering Cancer Center NCI Core Grant (P30 CA 008748) to FM; by AIRC, Milano, Italy (Investigator Grant – IG2017 #20052 to SB) and Italian Ministry of Education, Universities and Research (PRIN 2017 #2017PPS2X4_003 to SB). FM is also supported by the American Society of Hematology, the International Myeloma Foundation and The Society of Memorial Sloan Kettering Cancer Center. LA is supported by the Accelerator Award #A29374 through the CRUK Manchester Institute - AIRC partnership.

## Conflict of Interest

The authors declare that the research was conducted in the absence of any commercial or financial relationships that could be construed as a potential conflict of interest.

